# Virtual screening-driven repositioning of etoposide as CD44 antagonist in breast cancer cells

**DOI:** 10.18632/oncotarget.8180

**Published:** 2016-03-18

**Authors:** Charmina Aguirre-Alvarado, Aldo Segura-Cabrera, Inés Velázquez-Quesada, Miguel A. Hernández-Esquivel, Carlos A. García-Pérez, Sandra L. Guerrero-Rodríguez, Angel J. Ruiz, Andrea Rodríguez-Moreno, Sonia M. Pérez-Tapia, Marco A. Velasco-Velázquez

**Affiliations:** ^1^ Facultad de Medicina, Universidad Nacional Autónoma de México (UNAM), México D.F., México; ^2^ Red de Estudios Moleculares Avanzados, Instituto de Ecología (INECOL) A.C., Clúster Científico y Tecnológico Biomimic^®^, Xalapa Veracruz, México; ^3^ Unidad de Desarrollo e Investigación en Bioprocesos, Escuela Nacional de Ciencias Biológicas-IPN, México D.F., México; ^4^ Centro de Biotecnología Genómica-IPN, Reynosa, Tamaulipas, México

**Keywords:** etoposide, CD44, breast cancer, epithelial-mesenchymal transition, cancer stem cells

## Abstract

CD44 is a receptor for hyaluronan (HA) that promotes epithelial-to-mesenchymal transition (EMT), induces cancer stem cell (CSC) expansion, and favors metastasis. Thus, CD44 is a target for the development of antineoplastic agents. In order to repurpose drugs as CD44 antagonists, we performed consensus-docking studies using the HA-binding domain of CD44 and 11,421 molecules. Drugs that performed best in docking were examined in molecular dynamics simulations, identifying etoposide as a potential CD44 antagonist. Ligand competition and cell adhesion assays in MDA-MB-231 cells demonstrated that etoposide decreased cell binding to HA as effectively as a blocking antibody. Etoposide-treated MDA-MB-231 cells developed an epithelial morphology; increased their expression of E-cadherin; and reduced their levels of EMT-associated genes and cell migration. By gene expression analysis, etoposide reverted an EMT signature similarly to CD44 knockdown, whereas other topoisomerase II (TOP2) inhibitors did not. Moreover, etoposide decreased the proportion of CD44^+^/CD24^−^ cells, lowered chemoresistance, and blocked mammosphere formation. Our data indicate that etoposide blocks CD44 activation, impairing key cellular functions that drive malignancy, thus rendering it a candidate for further translational studies and a potential lead compound in the development of new CD44 antagonists.

## INTRODUCTION

Breast cancer is the most common noncutaneous cancer in women and the leading cause of cancer-related mortality in females worldwide. In 2012, over 1.6 million new cases and 520,000 breast cancer-caused deaths were reported [1]. Despite advances in its treatment, 20% to 30% of patients with early breast cancer will experience relapse with distant metastatic disease [2], necessitating new therapeutic strategies.

CD44 is a multidomain transmembrane glycoprotein receptor, the major physiological ligand of which is the glycosamine glycan hyaluronan (HA) [3]. In human breast cancer, the expression of CD44 and HA correlates with unfavorable clinical outcomes [4–7], highlighting their function in disease progression. Although several isoforms of CD44 are produced by alternative splicing, all of them share the HA binding site [3].

The expression of CD44 must switch from variant isoforms (CD44v) to the standard isoform (CD44s) in order to promote tumorigenicity and EMT [8]. In breast cancer cells, activation of CD44s upregulates the transcriptional repressor ZEB1, which binds the promoter of the splicing factor ESRP1, resulting in self-sustaining ZEB1 and CD44 expression [9]. Forced upregulation of HA induces EMT in human mammary epithelial cells [10, 11] and lowers E-cadherin levels and nuclear translocation of β-catenin in spontaneous tumors from MMTV-Neu mice [12]. Accordingly, CD44s expression increases the migratory and invasive capacity of breast cancer cells [13] and promotes survival under detached conditions in metastatic tumor cells [14] during the development of metastasis.

Moreover, EMT is linked to the acquisition of the breast CSC phenotype. Subpopulations of stem-like cells from patients express markers of cells that have undergone EMT [15, 16]. Induction of the EMT in mammary epithelial cells and breast cancer cells facilitates the acquisition of the breast CSC phenotype, increasing their clonogenic and tumorigenic abilities [17–20]. CD44 is expressed in breast CSCs [21], and CD44-mediated induction of EMT favors CSC self-renewal and maintenance [22, 23].

The binding of HA to CD44 activates GSK3β, which is required for the maintenance of CSC properties and the acquisition of a mesenchymal phenotype in CSCs that undergo EMT [24]. CD44 also activates the transcriptional regulator Nanog and c-Jun N-terminal kinase (JNK) in breast cancer cells. Whereas Nanog activation upregulates stem cell regulators, such as Rex1 and Sox2 [24, 25], JNK stimulation effects the production of the oncogenic miR-21 [26]. Conversely, CD44 knockdown attenuates TGFβ1-induced EMT [27]; downregulates stem cell markers [24]; and impairs tumorsphere formation, tumor growth, and metastatic ability [28].

In summary, CD44 activation by HA favors breast tumor progression by controlling EMT, CSC phenotype, and metastasis (reviewed in [22]), making the receptor a potential therapeutic target for anticancer drug development [29]. In the present work we aimed to reposition small-molecule drugs as CD44 antagonists by structure-based virtual screening (SBVS). By consensus docking, we identified compounds with potential HA-blocking ability among > 11,000 FDA-approved, withdrawn, and experimental drug molecules. A refinement of the docking results by molecular dynamics simulations indicated that etoposide was the best candidate for biological validation.

Functional evaluation was performed in MDA-MB-231 breast cancer cells that fit the basal subtype [30], in which CD44^+^/CD24^−^ cells are enriched [31] and no therapy is available. Etoposide decreased cell binding to HA in ligand competition studies and HA adhesion assays as effectively as an anti-CD44 blocking antibody. Exposure of MDA-MB-231 cells to etoposide for 24 h induced an epithelial morphology; increased the expression of downregulated genes, such as E-cadherin; and reduced the levels of EMT-associated genes and cell migration. At the evaluated etoposide concentrations these effects were unrelated to significant necrosis or apoptosis. Analysis of gene expression data from public databases showed that etoposide reverted a validated EMT signature in breast cancer cells [32]. The expression changes that were induced by other drugs that inhibit topoisomerase II (TOP2), the primary reported target of etoposide, were unrelated to the query signature, indicating that the effect of etoposide on EMT is independent of its activity as a TOP2 inhibitor.

Consistent with the link between EMT and stemness, etoposide diminished the proportion of cells with the CD44^+^/CD24^−^ phenotype, lowered chemoresistance, and blocked mammosphere formation. These data indicate that etoposide blocks CD44 activation, inhibiting cellular functions that drive malignancy, rendering it a candidate for further translational studies and a potential lead compound in the development of new CD44 antagonists.

## RESULTS

### Structure-based virtual screening (SBVS)

**Figure 1:**
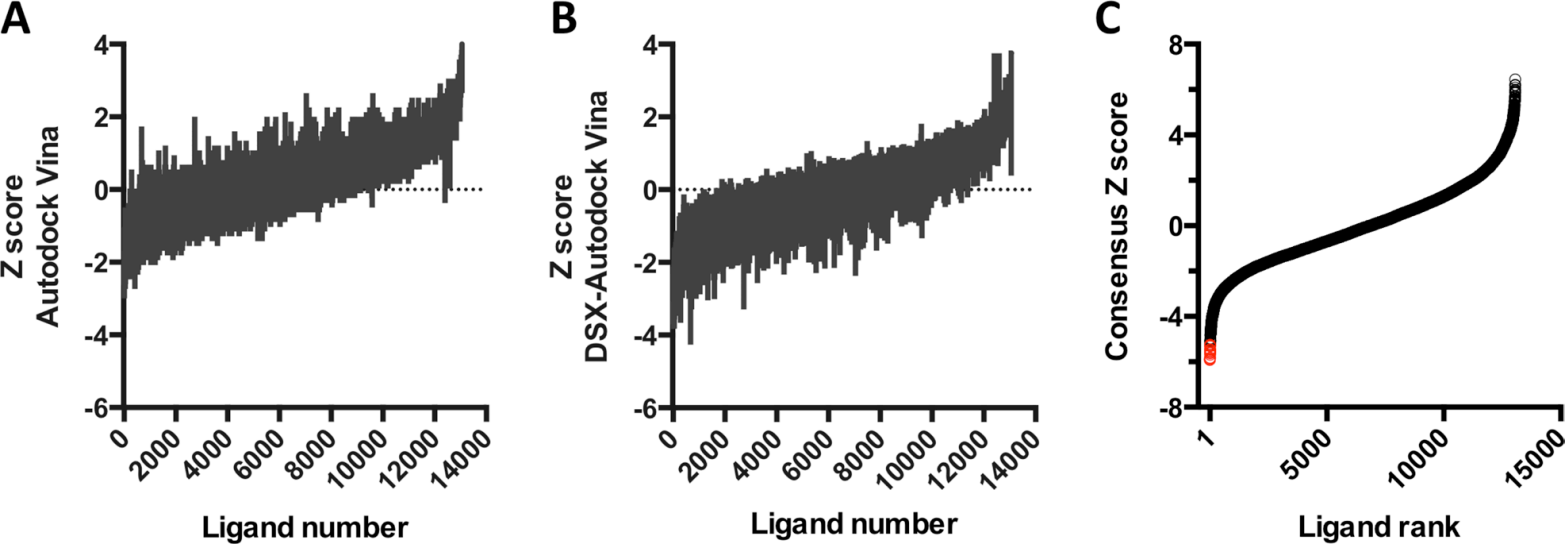
Virtual screen for CD44 antagonists (**A–B**) Z scores, calculated from the binding scores for each of the 13,066 structures using AutoDock Vina (A) or DSX_089 (B). (**C**) Ligands are ranked by consensus Z score, obtained by the sum of the Z scores from A and B. Red circles correspond to the ligands with the lowest predicted binding energies.

CD44 receptor binds to HA through its N-terminal HA-binding domain. Thus, we performed molecular docking using this domain as a receptor and 13,066 structures from 11,421 molecules from the In Man subset of the ZINC^12^ database as ligands. The docking scores that we obtained with AutoDock Vina and DSX_089 were normalized (Figure 1A–1B) and used to generate consensus Zscore values for each ligand. Based on the distribution of these values, we made unbiased identifications of 12 compounds with the best performance in the consensus docking protocol (Figure 1C).

**Figure 2:**
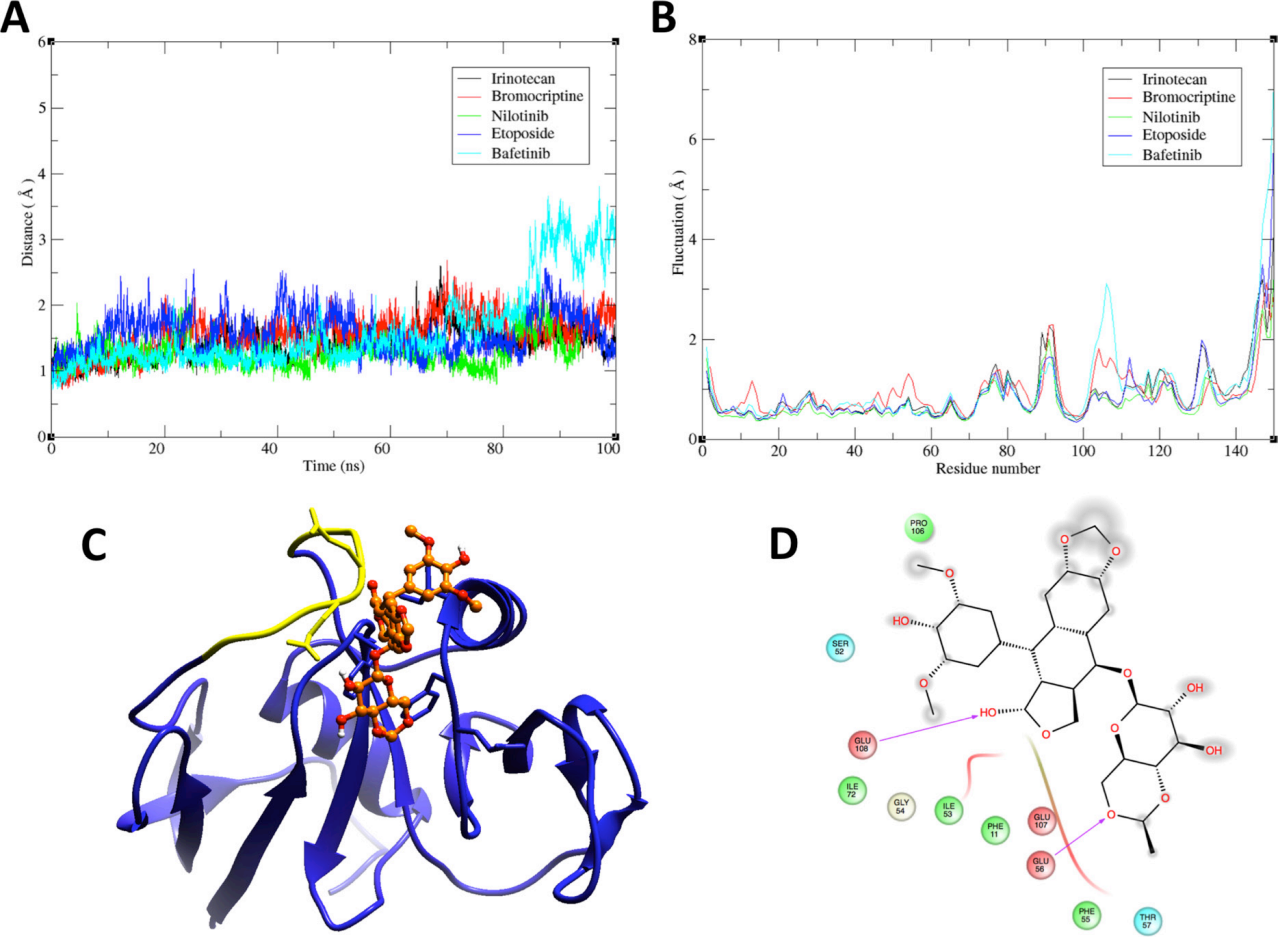
Molecular dynamics and molecular modeling of selected drugs (**A**) Root-mean-square deviation (RMSD)-versus-time plot for 5 drug-CD44 complexes. RMSD was calculated for the backbone atoms of the target protein (1UUH) during the MD simulation. (**B**) Root-mean-square fluctuation (RMSF) versus residue position for the backbone atoms of CD44. The color code is indicated in the top right panels in A and B. (**C**) 3D representation of the complex between etoposide (orange) and the HA-binding domain of CD44 (blue). Yellow region in CD44 corresponds to residues 100–110 on 1UUH, which showed the highest fluctuation in the RMSF analysis and includes residues mediating etoposide binding. (**D**) 2D representation of protein-ligand interactions. Residues in the binding site are represented as follows: acidic residues in red, polar residues in blue, hydrophobic residues in green, and glycine in beige. Magenta arrows indicate hydrogen bonding to backbone atoms. Grey “clouds” on ligand atoms indicate the solvent-exposed surface area.

We performed molecular dynamics simulations and binding free energy analysis with 5 candidate compounds: irinotecan, bromocriptine, nilotinib, etoposide, and bafetinib. By root-mean-square deviation (RMSD) analysis of the starting structure, we examined the stability of the 5 ligand-protein complexes (Figure 2A). Most complexes reached equilibrium at ~40 ns, except for the bafetinib-CD44 complex, in which the compound moved away from the HA binding site.

The analysis of the root-mean-square fluctuation (RMSF) per residue for the 5 systems is shown in Figure 2B. The chief backbone fluctuations occurred in the loop regions, particularly in the loop that outlined the residues that mediate HA binding (marked in yellow in Figure 2C). The bafetinib-CD44 complex showed the most drastic change in this loop, suggesting that this fluctuation is related to disassembly of the complex.

The binding free energies for the 5 protein-ligand systems were calculated using molecular mechanics -generalized Born surface area (MM-GBSA) method, and their values are compared in Table 1. The lowest free energy value corresponded to the etoposide-CD44 complex, indicating that etoposide was the best candidate for biological validation. Analysis of a representative snapshot of the clustering of the trajectory of the last 20 ns of stable MD simulation (Figure 2C) allowed us to define the types of interactions that governed drug-target binding. The interactions between molecules were predominantly mediated by van der Waals forces and hydrogen bonds (Figure 2D) that involved the Glu56 and Glu108 of the structure, corresponding to residues Glu75 and Glu127 in the primary structure of CD44.

**Table 1:** Calculated binding free energies (ΔG) for CD44-drug complexes

Ligand	Δ*G_ele_*	Δ*G_vdw_*	Δ*G_nonpol_*	Δ*G_pol_*	Δ*H*	*–T*Δ*S*	Δ*G_bind, calc_*
Irinotecan	−123.6783 (0.6)	−22.4702 (0.1)	−2.2311 (0.05)	132.9388 (0.6)	−15.4589 (0.1)	−13.8303 (1.4)	−1.6286
Bromocriptine	−13.5746 (0.1)	−21.9887 (0.01)	−2.6721 (0.01)	23.9434 (0.1)	−14.2921 (0.07)	−18.4837 (0.5)	4.1916
Nilotinib	−7.3423 (0.2)	−16.8807 (0.08)	−2.1595 (0.01)	16.9177 (0.2)	−9.4620 (0.1)	−21.1496 (0.7)	11.6876
**Etoposide**	**−9.6765 (0.5)**	**−20.2363 (0.2)**	**−2.5537 (0.03)**	**19.9953 (0.6)**	**−12.4750 (0.1)**	**0.6331 (1.9)**	**−13.1081**
Bafetinib	−12.9836 (0.5)	−29.4973 (0.1)	−19.6111 (0.01)	41.3551 (0.4)	−1.1204 (0.1)	−20.6973 (3.9)	19.5769

The values in parenthesis represent the standard error of mean.

### Etoposide inhibits the binding of CD44^+^ breast cancer cells to HA

**Figure 3:**
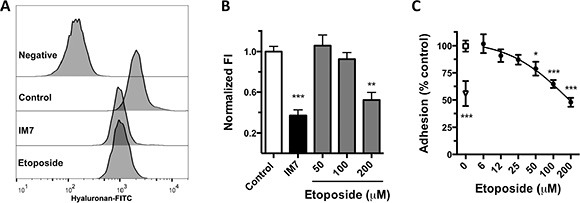
Inhibition of HA-CD44 binding by etoposide (**A**) Flow cytometry histograms of HA-FITC binding to MDA-MB-231 control cells (0.2% DMSO) or cells treated with anti-CD44 (mAb IM7) or 200 μM etoposide. Negative fluorescence consists of cells incubated with nonfluorescent HA. (**B**) Quantification of normalized fluorescence index (FI; see “Methods”) from 5 independent experiments (means ± SEM). **P < 0.01, ***P < 0.001 by Bonferroni's multiple comparisons test. (**C**) Cell adhesion of MDA-MB-231 cells to HA-coated microplates. Cells were treated with 0.2% DMSO (□), various concentrations of etoposide (·), or IM7 antibody (Δ). Data are means ± SEM from 3 independent experiments. *P < 0.05, ***P < 0.001 by Bonferroni's multiple comparisons test.

We analyzed the *in vitro* activity of etoposide as a CD44 antagonist using MDA-MB-231 breast cancer cells, > 95% of which express high levels of CD44 [33]. By flow cytometry, we determine the ability of etoposide to inhibit the binding of CD44 to fluorescein isothiocyanate-coupled HA (HA-FITC). Over 95% of vehicle-treated cells bound the ligand, showing positive fluorescence. Using a blocking monoclonal antibody (clone IM-7) that targets the HA-binding domain of CD44, we found that HA-FITC binding to MDA-MB-231 cells is mediated in part by CD44. Preincubation of MDA-MB-231 cells with etoposide (200 μM) for 15 min significantly reduced the fluorescence index to 52.2 ± 13.7% of that of vehicle-treated cells. The inhibition of binding that was induced by IM-7 did not differ significantly from that by 200 μM etoposide, indicating that etoposide is as effective as IM-7 in blocking CD44-HA binding (Figure 3A–3B).

Further, we analyzed the capacity of etoposide to inhibit HA-induced cell adhesion. In static adhesion assays, etoposide significantly decreased the adhesion of MDA-MB-231 cells to a layer of HA dose-dependently from 50 μM to 47.8 ± 13.2% of control at 200 μM (Figure 3C). These results indicate that etoposide inhibits HA binding to CD44 and CD44-activated cell functions, supporting its function as a CD44 antagonist.

### Etoposide reverts EMT without inducing cell death

**Figure 4:**
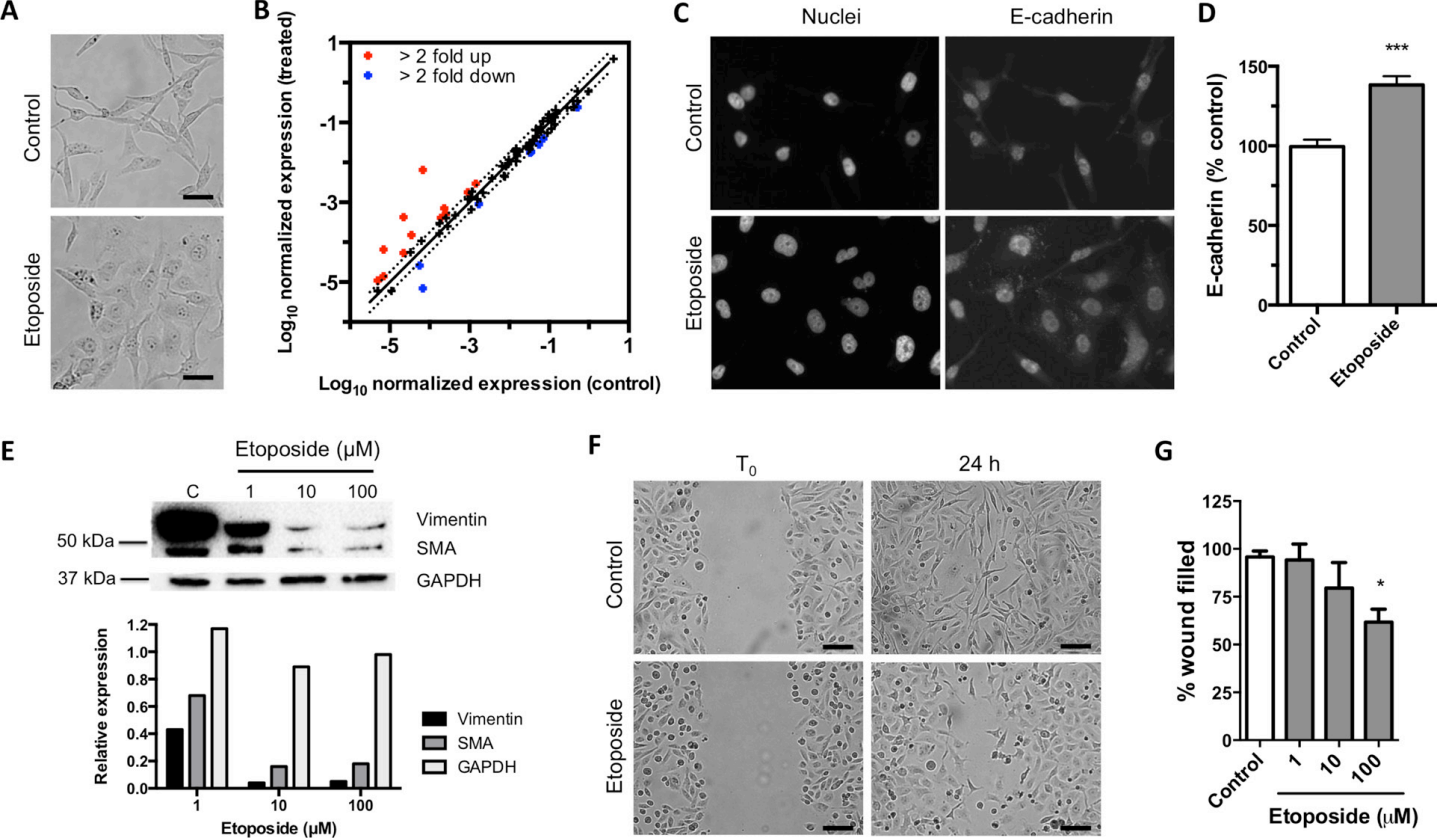
Exposition to etoposide reverts EMT (**A**) Representative images of MDA-MB-231 cell morphology after treatment with 0.2% DMSO (control) or 10 μM etoposide for 24 h. Scale bars = 50 μm. (**B**) Comparison of expression of EMT-related genes in cells treated with 10 μM etoposide versus control cells. Dots in red represent genes upregulated ≥ 2-fold; blue dots represent genes downregulated ≥ 2-fold compared with control. (**C**) Representative immunostains with anti-E cadherin of MDA-MB-231 cells treated with 0.2% DMSO (control) or 10 μM etoposide for 24 h. (**D**) Quantification of the staining signals in C. Bars represent normalized E-cadherin fluorescence intensity per cell (mean ± SEM). Over 200 cells were analyzed per condition (***P < 0.001, Student's *t*-test). (**E**) Western blot of control and etoposide-treated cells (upper panel) and densitometry analysis of the western blot bands (lower panel). (**F**) Representative micrographs from wound healing assay of MDA-MB-231 cells treated with 0.2% DMSO (control) or 100 μM etoposide. Scale bars = 100 μm. (**G**) Quantification of 4 independent wound healing assays of MDA-MB-231 cells treated with 0.2% DMSO or various concentrations of etoposide. Bars represent the percentage of wound filled after 24 h of treatment versus control (means ± SEM). *P < 0.05, ***P < 0.001 by Bonferroni's multiple comparisons test.

Etoposide reshaped the predominantly mesenchymal morphology of MDA-MB-231 cells to a more epithelial phenotype (Figure 4A). Given these changes and the reported function of CD44 in controlling EMT, we compared the expression of 84 EMT-related genes in control and etoposide-treated cells by qRT-PCR (Figure 4B). Treatment with 10 μM etoposide for 24 h induced the differential expression of EMT-related genes in MDA-MB-231 cells. In etoposide-treated cells, 12 genes rose ≥ 2-fold (BMP7, CDH1, COL3A1, COL5A2, ERBB3, FOXC2, IL1RN, KRT14, MMP3, SNAI3, VCAN, and WNT11), whereas 9 were downregulated ≥ 2-fold (COL1A2, EGFR, ESR1, MMP2, NODAL, PTK2, SERPINE1, SNAI2, and STEAP1) compared with the control (Figure 4B). By western blot and immunofluorescence, etoposide reverted the loss of the epithelial differentiation protein E-cadherin (Figure 4C–4D) and downregulated vimentin and SMA in MDA-MB-231 cells (Figure 4E). We also tested the ability of etoposide to modify mesenchymal behavior by cell migration assay. Etoposide reduced MDA-MB-231 cell migration (Figure 4F–4G). These effects were independent of the cytotoxic effect of etoposide. At the concentration that we used in the assays shown in Figure 4A–4D (10 μM), etoposide did not induce significant apoptosis or necrosis (Supplementary Figure 1A) and did not change the number of viable cells up to 200 μM (Supplementary Figure 1B). These data indicate that etoposide partially reverts the mesenchymal phenotype of MDA-MB-231 cells without altering cell viability.

### Etoposide, but not other TOP2 inhibitors, reverts an EMT signature in breast cancer cells

The function of TOP2 inhibition in the etoposide -induced phenotypic changes was evaluated using the LINCS L1000 dataset [34]. We analyzed the changes in expression due to the TOP2 inhibitors and compared them with a signature that was generated by the induction of EMT in human mammary epithelial cells [32]. Because there were no available data on etoposide-treated basal breast cancer cells, we used MCF-7 cells.

The EMT signature correlated negatively with CD44 knockdown-induced gene expression (Table 2), supporting the function of CD44 in promoting EMT. Etoposide had a negative enrichment score in the database, whereas the expression changes that were induced by the TOP2 inhibitors ellipticine, mitoxantrone, doxorubicin, and daunorubicin were unrelated to the query signature (Table 2). These results indicate that EMT reversion in breast cancer cells can be effected by etoposide but not other TOP2 inhibitors and that etoposide reverts the EMT signature as effectively as knocking down CD44.

**Table 2:** Chemical genomics analysis to prioritize compounds in MCF-7 cell line

Perturbagen	LINCS Score
shCD44	−89.3
Etoposide	−98.1
Ellipticine	0
Mitoxantrone	0
Doxorubicin	0
Daunorubicin	0

### Etoposide decreases the CSC population

**Figure 5:**
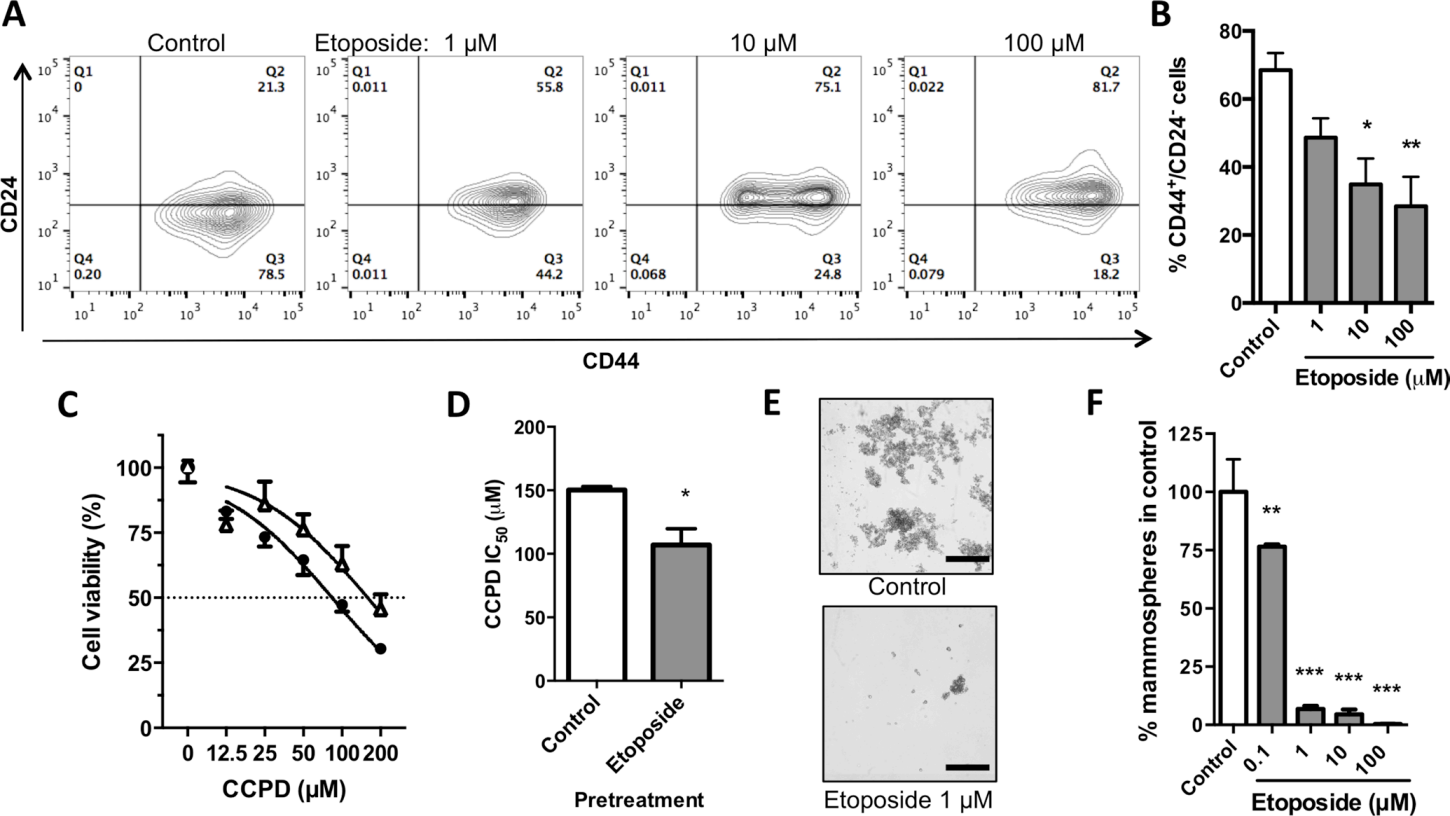
Effects of etoposide on stem-like properties of MDA-MB-231 breast cancer cells (**A**) Flow cytometric analysis of CD44 and CD24 expression in MDA-MB-231 cells treated with etoposide for 24 h. (**B**) Quantification of percentage of CD44^+^/CD24^−^ cells from 4 independent experiments as depicted in A. Data are means ± SEM. *P < 0.05, **P < 0.01 by Bonferroni's multiple comparisons test. (**C**) Normalized cell viability of MDA-MB-231 cells exposed to CCPD after being treated for 24 h with 10 μM etoposide (Δ) or vehicle (·; 0.2% DMSO). Data are means ± SEM, *n* = 6. (**D**) Bar graph comparing IC_50_ values for CCPD after pretreatment with etoposide (10 μM) or vehicle control from 2 independent experiments as represented in C. *P < 0.05, Student's *t*-test. (**E**) Representative images of mammospheres formed by MDA-MB-231 cells cultured in the presence of 0.2% DMSO (control, upper panel) or 1 μM etoposide (lower panel) for 7 days. Bar scale is 200 μM. (**F**) Bar graph of normalized number of mammospheres formed by MDA-MB-231 cells treated with various concentrations of etoposide for 7 days. Data are means ± SEM from 3 independent experiments; **P < 0.01, ***P < 0.001 by Bonferroni's multiple comparisons test.

Based on the function of CD44 as a CSC marker for breast cancer cells and the link between EMT and stemness, we determined the effects of etoposide on several CSC features. Etoposide (10 μM) significantly decreased the percentage of CD44^+^/CD24^−^ cells to 34.90 ± 13.18% versus 68.53 ± 9.93% in vehicle-treated (control) MDA-MB-231 cells (Figure 5A–5B). In normal and neoplastic mammary epithelial cells, EMT induction enlarged the CSC pool [17], which is associated with greater drug resistance. Thus, we evaluated the ability of etoposide to alter the sensitivity to the cytotoxic drug cisplatin (CCPD). Pretreatment of MDA-MB-231 cells for 24 h with etoposide decreased the CCPD half maximal inhibitory concentration (IC_50_) from 150.30 ± 4.67 μM to 107 ± 22.25 μM, indicating that the EMT reversion increases the sensitivity to cytotoxic drugs (Figure 5C–5D). In mammoesphere assays etoposide significantly lowered the formation of spheres at concentrations as low as 0.1 μM (Figure 5E–5F). These data indicate that etoposide shrinks the pool of CSCs in MDA-MB-231 breast cancer cells.

## DISCUSSION AND CONCLUSIONS

In this study, using SBVS, we predicted that the TOP2 inhibitor etoposide could bind the hyaluronan-binding domain of CD44. The region of the receptor used in our analyses contains the essential amino acids for HA binding (Arg41, Tyr42, Arg78, and Tyr79) and the functionally important residues Lys38, Lys68, Asn100, Asn101, and Tyr105 [35]. Mapping of these amino acids to the structure of CD44 has shown that all but Lys68 form a contiguous linear patch [36]. Our MD results demonstrate that etoposide binds stably to the target structure interacting with Glu75 and Glu127 in CD44, which also reside in the surface patch that is responsible for HA binding.

*In vitro*, we found that etoposide decreases the binding of MDA-MB-231 breast cancer cells to HA as effectively as anti-CD44 in ligand competition and cell adhesion assays, indicating that etoposide antagonizes CD44 physiologically. Because the selection of antagonist was based on the structure of the protein, etoposide should be able to block CD44 independently of the cellular context.

CD44 activation by HA in basal breast cancer cells promotes additional CD44 expression, HA synthesis, and activation of signaling pathways that control EMT and stemness [28, 37]. We found that 24-h exposure to concentrations as low as 10 μM etoposide induced phenotypic changes in MDA-MB-231 cells, reverting the mesenchymal phenotype, reducing the proportion of CD44^+^/CD24^−^ cells, inhibiting tumorsphere formation, and increasing sensitivity to CCPD. Accordingly, CD44 downregulation by shRNA decreases migration, invasion, MMP expression, tumorsphere formation, tumor growth, and metastatic ability in breast cancer cells [28, 38]. Consistent with our findings, etoposide is selectively toxic to mesenchymally transdifferentiated mammary epithelial cells, which have features of CSCs [39].

The mechanism of action of etoposide as a cytotoxic drug is the inhibition of TOP2. Etoposide induces the generation of double-stranded DNA breaks (DSDBs) by covalent trapping of TOP2 into DNA cleavage complexes [40]. In cancer cells with wild-type p53, DSDBs lead to ATM-dependent cell death [41, 42]. However, in cancer cells with mutant p53, such as MDA-MB-231 [43, 44], the cytotoxic effect of etoposide is mitigated [45]. Exposure of MDA-MB-231 cells to 50 μM etoposide for 24 h effects apoptosis in less than 5% of cells [46, 47]. Similarly, we noted that etoposide does not induce significant cytotoxicity or apoptosis in MDA-MB-231 cells, indicating that the phenotypic and functional changes that we observed are independent of activated cell death pathways. Further, the data that we generated with the LINCS L100 platform showed that etoposide, but not other TOP2 inhibitors, reverts a validated EMT signature in MCF-7 cells, suggesting that etoposide-mediated EMT reversion is also achieved in other breast cancer cells and supporting the hypothesis that this effect is independent of TOP2 inhibition.

Repositioning of etoposide as a CD44 antagonist is a proof of concept that the combination of target-based *in silico* screening and repositioning can quickly identify anticancer drug candidates. Although SBVS has been widely applied in early-stage drug discovery of anticancer agents [48], its combination with a repurposing strategy is uncommon. One of the few available examples of this approach was reported by Shi and colleagues [49], who used a docking method with > 4000 FDA-approved drugs to identify the antipsychotic drug fluspirilene as a CDK2 inhibitor, which was found to be effective in preclinical models of hepatocellular carcinoma.

Although further mechanistic analyses are required to understand the CD44-mediated effects of etoposide, its has been reported that oral etoposide as single-agent induces objective response rate in metastatic breast cancer patients previously exposed to chemotherapy [50–52]. Likewise the combined administration of bevacizumab with etoposide and cisplatin exhibited promising anti-angiogenical efficacy in patients with leptomeningeal carcinomatosis originating from breast cancer [53]. In addition, etoposide can become a lead compound in the development of CD44 blocking agents. Recently, it was shown that the etoposide analog C-10 reduces cell viability, and cell migration of breast cancer cell lines through expression of microRNAs-15, 16, 17 and 221 and their target protein STAT3 [54]. Thus, etoposide might engender new therapies that, in combination with current agents, prevent relapse and metastasis and increase breast cancer patient survival.

## MATERIALS AND METHODS

### Molecular docking

The X-ray diffracted structure of the human CD44 hyaluronan-binding domain has been reported [36] and was obtained from Protein Data Bank (PDB code 1UUH). The druggability of protein pockets was assessed using the DoGSiteScorer server tool (Hamburg University, Germany) [55]. A library of 13,066 structures from 11,421 molecules were retrieved from the ZINC In Man dataset (FDA-approved, withdrawn, and experimental drugs) from subsets of the ZINC^12^ database (University of California, San Francisco) [56]. Docking was performed with residues 13 to 102 of PDB code 1UUH. Auto-DockTools was used to add the Gasteiger charges and polar hydrogens to CD44 and the drugs. The grid box in the hyaluronan link module of CD44 was set to 40Å × 40Å × 40Å with 0.375-Å spacing to perform docking with AutoDock Vina 1.1.2 [57]. The scoring results of AutoDock Vina were re-evaluted with DSX_089 [58] to increase the reliability and accuracy of the antagonist selection. We normalized the scores for each algorithm, calculating the Zscore as follows

Zscore = (*fi-*μ*)/*σ

where *fi* is the scoring value of a certain scoring function, μ is the mean value, and σ is the standard deviation of the scoring function. The consensus score was the sum of Zscores.

### Molecular dynamics

MD simulation was performed to evaluate the stability, folding, conformational changes, and dynamic behavior of ligands that interacted with the CD44 hyaluronan-binding domain (PDB code 1UUH) using Amber ff99SB force fields and the Amber 12 package [59]. During the MD simulations, all systems were solvated using the TIP4P [60] water model in a periodic box, followed by the addition of Na^+^ and Cl^−^ counterions to neutralize the systems. Before the MD simulations, energy minimization and equilibration of the system were performed under specified pressures and temperatures using AmberTools. Finally, the MD simulations proceeded for 100 ns at constant temperature (300 K) and pressure (1 atm). Trajectory snapshots were taken every picosecond for the structural analysis. Root mean square deviations (RMSDs) and backbone atomic fluctuations (RMSFs) in the docked complex were analyzed in AmberTools 12. The binding free energy (ΔG) between ligands and CD44 was calculated using the MM-GBSA method as implemented in AmberTools 12 [61].

### Cell culture

The MDA-MB-231 breast adenocarcinoma cell line was obtained from ATTC and cultured in L-15 medium (ATCC) that was supplemented with 10% fetal bovine serum (FBS; GIBCO) at 37°C.

### Chemicals

Etoposide (Sigma-Aldrich) was prepared as a 50 mM stock solution in dimethyl sulfoxide (DMSO) and stored at −70°C until use.

### HA binding assays

We measured the binding of HA-FITC (Sigma-Aldrich) to MDA-MB-231 cells by flow cytometry as reported [62, 63]. Briefly, cells were preincubated for 15 min at RT with anti-CD44 (mAb IM-7) or etoposide before being exposed to HA-FITC (20 μg/mL) for 30 min. The mean fluorescence intensity of cells was analyzed by flow cytometry (FACSAria III, BD Biosciences o Attune NxT, Life Technologies). Cells that were incubated with unlabeled HA were used to set the negative florescence signal. Data were analyzed with FlowJo, version 8.7 (Tree Star, Inc.), and the mean fluorescence intensities (MFIs) were used to calculate a fluorescence index: MIF_x_-MFI_negative_/MIF_control_-MFI_negative_.

### Cell adhesion assays

Cell adhesion was evaluated as reported [64]. HA (Sigma-Aldrich) was adsorbed overnight onto various wells of a 96-well microplate (20 μg HA/cm^2^). Before the assay, the wells were washed and blocked with heat-denatured bovine serum albumin (BSA fraction V; Sigma-Aldrich) that was diluted in PBS (10 mg/mL). Some wells were coated only with BSA to estimate nonspecific adhesion. Cells were collected by nonenzymatic methods and preincubated for 15 min with anti-CD44 (IM-7) or etoposide in medium with 0.5% FBS. Then, 30,000 cells were added to each well and allowed to adhere for 45 min at 37°C. Nonadherent cells were removed by washing and the remaining cells were fixed and quantified by crystal violet staining. HA-specific adhesion was normalized to untreated cells.

### Cell viability assay

Effects on cell viability were estimated using the MTS [3-(4,5-dimethylthiazol -2-yl)-5-(3-carboxymethoxyphenyl)-2-(4-sulfophenyl)-2H- tetrazolium] assay (Promega) as reported [65]. The amount of reduced tetrazolium salt, which is proportional to the number of viable cells, was measured spectrophotometrically at 490 nm. The experiments were performed in sextuplicate in 96-well microplates.

### Flow cytometric analysis of cell death

Drug-treated cells were collected and stained simultaneously with annexin V-APC FITC (Life Technologies) and 7-amino-actinomycin (7-AAD; BD Pharmigen) as reported [66]. As positive controls for necrosis and apoptosis, we included heat-shocked (60°C, 60 min) and camptothecin-treated (100 ng/mL, 24 h) cells, respectively. A minimum of 10,000 cells were acquired in Attune NxT (Life Technologies), and the data were analyzed with FlowJo, version 8.7 (Tree Star Inc.).

### qRT-PCR

We used the Human Epithelial to Mesenchymal Transition RT^2^ Profiler PCR Array (Qiagen PAHS-090Z) to analyze the effects of etoposide on the expression of 84 EMT-related genes. Total RNA was extracted from 10^6^ cells after 24 h of treatment with or without etoposide (10 μM) using the RNeasy^®^ Mini Kit (Qiagen) per the manufacturer's instructions. The purity and quality of isolated RNA were determined by measuring the ratio of absorbance values at 260 and 280 nm and by agarose gel electrophoresis. cDNA was synthetized from 5 μg of total RNA using the RT^2^ First Strand Kit (Qiagen), which includes the additional removal of genomic DNA from the sample and a specific control for reverse-transcription. qRT-PCR was performed using the PCR Array on an ABI7500 FAST (Applied Biosystems) per the manufacturer's instructions. Genes with changes in expression greater than 2-fold were selected after normalizing the ΔΔC_T_ value of each gene to that of constitutively expressed genes with RT^2^ Profiler PCR Array Data Analysis Webportal (Qiagen), available at pcrdataanalysis.sabiosciences.com.

### Immunofluorescence

MDA-MB-231 cells were seeded in LabTek chambers (Nunc) and treated with 10 μM etoposide or the corresponding vehicle for 24 h. Then, the cells were fixed with 4% paraformaldehyde at room temperature (RT) and permeabilized with cold methanol at −20°C. Nonspecific sites were blocked with 5% FCS for 20 min. Cells were incubated with antibodies against E-cadherin (Santa Cruz Biotechnology) at 4°C overnight. After a wash step, the cells were incubated with anti-rabbit coupled to Alexa-488 (Invitrogen MP) for 1 h at RT. To stain nuclei, cells were treated with RNAse A (2 mg/ml) for 1 h at 37°C and then incubated with propidium iodide (50 μg/ml) for 5 min at RT. Preparations were analyzed with the Cytation^™^ 5 image multimode reader (Biotek). Fluorescence intensity per cell was resolved using imageJ.

### Western blot

Etoposide-treated or control MDA-MB-231 cells were lysed in RIPA buffer (50 mM Tris-HCl, 1% NP-40, 0.5% sodium deoxycolate, 0.1% SDS, 150 mM NaCl, 2 mM EDTA, 50 mM NaF) that was supplemented with protease inhibitor cocktail (Roche). Lysates were centrifuged for 15 min at 12,000 rpm at 4°C, and protein concentrations in the supernatants were determined using the Pierce BCA Protein Assay Kit (Thermo Fisher Scientific). Samples (30 μg of total protein) were separated by SDS-PAGE and electroblotted onto PVDF membranes. The membranes were blocked with 5% nonfat milk in 1× TTBS (1× TBS and 0.1% Tween 20) for 1 h at RT. Membranes were incubated with the primary antibodies (EMT WB cocktail, Abcam), diluted in 5% milk/PBS, overnight at 4°C. After being washed, the membranes were incubated with HRP-conjugated secondary antibody cocktail. Protein bands were detected using Pierce ECL Western Blotting Substrate (Thermo Fisher Scientific) on the ChemiDoc Imaging System (BIO-RAD). Images were analyzed in Image Lab (BIO-RAD).

### Wound healing assay

Experimental wounds were created by dragging a pipette tip across confluent cell cultures. The cultures were rinsed twice with PBS to remove unattached cells, and media (0.5% FBS) that contained the drug or corresponding vehicle was added. The same fields were photographed immediately (*t* = 0) and 24 h later under an inverted microscope (IX51 microscope and DP73 camera, Olympus). Two wounds were sampled for each specimen. The gap distance was quantified using CellSense (Olympus). Assays were repeated in 4 independent experiments.

### Flow cytometry

Cell surface expression of CD44 and CD24 was analyzed, based on previous reports [67]. Briefly, drug-treated cells were harvested with TrypLE Select Enzyme (Gibco) and simultaneously stained with anti-CD44-APC (clone G44-26, BD Pharmigen) and anti-CD24-PE (clone ML5, BD Pharmigen) in PBS that contained 10% FBS. Isotype controls were used to establish the negative fluorescence signal. Samples were analyzed on a FACS Aria III (BD Biosciences) or Attune NxT (Life Technologies), and the data were analyzed with FlowJo, version 8.7 (Tree Star Inc.).

### Mammosphere assays

Single-cell suspensions were plated in 6-well ultralow-attachment plates (Corning Costar) at 1000 cells/mL using MammoCult medium plus growth factors (StemCell Technologies) [68]. Cultures were grown for 7 days, with drug the drug being added at 0 h and 72 h. Mammospheres were photographed under an IX51 microscope with a DP73 camera (Olympus). Spheres > 80 μm were counted using NIS Elements Br (Nikon), and the total number of spheres on treated cultures was normalized against that in vehicle-treated cultures.

### LINCS L1000 analysis

We performed a chemical genomics analysis using the L1000 signatures from the National Institutes of Health (NIH)-funded Library of Integrated Network-based Cellular Signatures (LINCS: http://www.lincscloud.org/) [34]. The top 100 upregulated and downregulated genes from the GSE9691 dataset of the GEO-NCBI database were used to build 2 gene lists. The GSE9691 dataset corresponds to “EMT core signature,” as reported by Taube et al. [32]. These gene lists were used as input to match to L1000 signatures from LINCS. We were searching for candidate drugs that were predicted by our docking protocol to inhibit CD44 if they were negatively associated (negative LINCS score) with the EMT core signature in LINCS, thus indicating that they can induce EMT reversion in cells.

The LINCS score indicates how well a particular perturbagen is connected to the query in the given cell lines. The score ranges from −100 (complete anticonnection) to 100 (complete connection). Thus, positive values indicate that the perturbation effects a similar signature to the query, and negative values indicate that it yields a signature that is opposite to that of the query. Scores with a magnitude of greater than +/− 90 correspond to significant connections.

### Statistical analysis

Results from the biological assays were compared by ANOVA, followed by Bonferroni's multiple comparisons test (binding and cell adhesion, wound healing, receptor expression, and mammosphere assays). E-cadherin expression was analyzed by student′s *t*-test. All tests were performed with Prism 6.0 (GraphPad), and the null hypothesis was rejected when *P* < 0.05.

## SUPPLEMENTARY MATERIALS FIGURE


