# Supplementary material for: Virtual screening-driven repositioning of etoposide as CD44 antagonist in breast cancer cells
Source: Oncotarget. 2016 Mar 18;7(17):23772–84. doi: 10.18632/oncotarget.8180 (PMC5029662; doi:10.18632/oncotarget.8180)
Supplement: Supplementary file 1 [file oncotarget-07-23772-s001.pdf]

Virtual screening-driven repositioning of etoposide as CD44 antagonist in breast cancer cells

Supplementary Materials

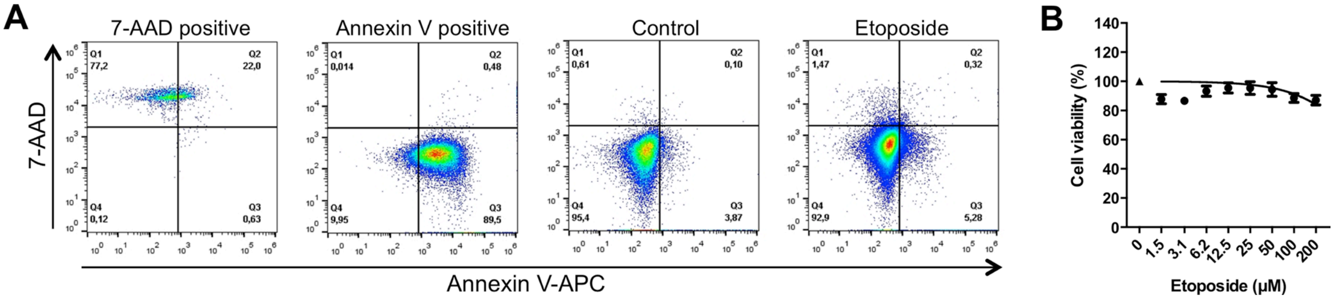

Supplementary Figure S1: Effect of etoposide on cell death. (**A**) Flow cytometric determination of apoptosis and necrosis in MDA-MB-231 cells treated with DMSO 0.2% (control) or etoposide (10 µM) for 24 h. Heat-shocked (7-AAD positive) or camptothecin-treated (Annexin V positive) cells were used as positive controls for necrosis and apoptosis, respectively. (**B**) MTS assay in MDA-MB-231 cells exposed for 24 to different concentrations of etoposide.
